# On the role of transforming growth factor-β in the growth inhibitory effects of retinoic acid in human pancreatic cancer cells

**DOI:** 10.1186/1476-4598-6-82

**Published:** 2007-12-24

**Authors:** Brahmchetna Singh, Richard F Murphy, Xian-Zhong Ding, Alexandra B Roginsky, Richard H Bell, Thomas E Adrian

**Affiliations:** 1 Department of Surgery and Robert H Lurie Comprehensive Cancer Center, Northwestern University Feinberg School of Medicine, Chicago, IL, USA; 2 Department of Biomedical Sciences, Creighton University, Omaha, NE, USA; 3 Department of Physiology, Faculty of Medicine and Health Sciences, United Arab Emirates University, Al Ain, UAE

## Abstract

**Background:**

Retinoids are potent growth inhibitory and differentiating agents in a variety of cancer cell types. We have shown that retinoids induce growth arrest in all pancreatic cancer cell lines studied, regardless of their p53 and differentiation status. However, the mechanism of growth inhibition is not known. Since TGF-β2 is markedly induced by retinoids in other cancers and mediates MUC4 expression in pancreatic cancer cells, we investigated the role of TGF-β in retinoic acid-mediated growth inhibition in pancreatic cancer cells.

**Results:**

Retinoic acid markedly inhibited proliferation of two cell lines (Capan-2 and Hs766T) in a concentration and time-dependent manner. Retinoic acid increased TGF-β2 mRNA content and secretion of the active and latent forms of TGF-β2 (measured by ELISA and bioassay). The concentrations of active and TGF-β2 secreted in response to 0.1 – 10 μM retinoic acid were between 1–5 pM. TGF-β2 concentrations within this range also inhibited proliferation. A TGF-β neutralizing antibody blocked the growth inhibitory effects of retinoic acid in Capan-2 cells and partially inhibitory the effects in Hs766T cells.

**Conclusion:**

These findings indicate that TGF-β can cause growth inhibition of pancreatic cancer cells, in a p53-independent manner. Furthermore, it demonstrates the fundamental role of TGF-β in growth inhibition in response to retinoic acid treatment is preserved *in vitro*.

## Background

Pancreatic adenocarcinoma is currently the fourth leading cause of cancer death in the United States [1] and histologically constitutes 90% of pancreatic tumors. The diagnosis of pancreatic cancer is usually established at a locally advanced or metastatic stage. Lack of effective treatments and resistance to conventional therapy contribute to an extremely poor prognosis [1,2]. Vitamin A (retinol) and its natural derivatives (retinoids) are involved in several important physiological processes such as reproduction, cell proliferation, differentiation and embryonic development [3]. Human clinical trials have demonstrated retinoic acid suppresses development of oral premalignant lesions, head and neck and skin cancer [4]. Patients with acute promyelocytic leukemia are particularly sensitive to retinoic acid treatment. Their response rates are in the range of 90% with retinoic acid monotherapy [5]. Retinoic acid can arrest growth in a number of different cell types [6,7].

One member of the retinoic acid receptor family (collectively called RAR) recognize two natural stereo isomers of retinoic acid, all-*trans *retinoic acid (ATRA), and 9-*cis *retinoic acid. In contrast, another receptor family (collectively called RXR) only recognizes 9-*cis *retinoic acid. The pleiotropic effects of retinoids are mediated with each individual receptor sub-type controlling distinct gene expression patterns important for cell growth and differentiation. Target genes include transcription factors, enzymes, cytokines and growth factors [8]. In some cancer cell types, retinoic acid-mediated growth inhibition is associated with reduced expression of transcription factors such as c-myc, c-myb, p53, pRB and also decreased expression of epidermal growth factor receptor [4]. However, the factors directly involved in mediating the anti-proliferative effects of retinoids have so far not been elucidated.

It has been previously demonstrated, that by optimizing the treatment conditions, a broad panel of pancreatic cancer cell lines that were reported to be resistant to retinoic acid were sensitized to the anti-proliferative effects and differentiation induction by retinoic acid *in vitro *and *in vivo *[9]. Retinoic acid also induces apoptosis in pancreatic cancer cells [10]. The purpose of the present study was to examine TGFβ as a likely candidate in mediating the growth inhibitory effects of retinoic acid in pancreatic cancer cells.

Members of the transforming growth factor-β (TGF-β) superfamily are known to potently inhibit the proliferation of many epithelial cell types [11]. TGF-β is secreted in a latent inactive complex in association with the latency associated peptide (LAP). Latent TGF-β (LTGF) is bound to additional high molecular weight proteins that are associated with LAP. The mechanisms of TGF-β activation include, proteolysis, enzymatic deglycosylation, acid-treatment, ROS and radiation [12]. Acid treatment of the conditioned media (CM) activates latent TGF-β, probably by denaturing the LAP either by conformational change or by disturbing the interaction between LAP and TGF-β [12]. TGF-β signaling is initiated by binding to the type I and type II cell-surface receptors, both of which are serine/threonine kinases. The Smad anchor for receptor activation (SARA), which is a membrane-associated protein, escorts unphosphorylated Smad2 and Smad 3 to the receptor, which in turn phosphorylates these Smad proteins [13]. These then associate with Smad4 and enter the nucleus and, together with other transcription factors, activate transcription of various target genes [14]. The impact of the loss of components of TGF-β signaling is seen in pancreatic and colon cancer. In pancreatic cancer, the DPC-4 (deleted in pancreatic Cancer-4/Smad-4) gene is inactivated in by homozygous deletion or intragenic mutation in 50% of cases [15]. This might be expected to abolish TGF-β signaling, however, recent studies have clearly demonstrated Smad-4 independent signaling pathways in pancreatic cancer cells as well as in several other systems [16-19]. TGF-β and retinoic acid are associated in both a physiological and pathological context [20,21]. Both TGF-β and retinoic acid inhibit the growth of retinal pigment epithelial cells, bone marrow progenitor cells, and human cervical and breast cancer cells [22-25]. TGF-β2 mediates the expression of MUC-4 in retinoic acid treated pancreatic cancer cells [26]. The TGF-β2 isoform is selectively induced by retinoic acid in keratinocytes [27]. Retinoic acid also induces the activation of latent TGF-β and its receptor [15,21,28-31]. Furthermore, neutralizing antibodies against TGF-β can block retinoic acid-induced growth inhibition of several types of cells including keratinocytes and leukemia cells. [32,33].

Based on the previous studies establishing a relationship between retinoic acid and TGF-β, this growth factor appeared to be a likely candidate to mediate the growth inhibitory effects of retinoic acid in pancreatic cancer. This role was investigated in the following studies.

## Results

### Growth Studies with retinoic acid

**Figure 1:**
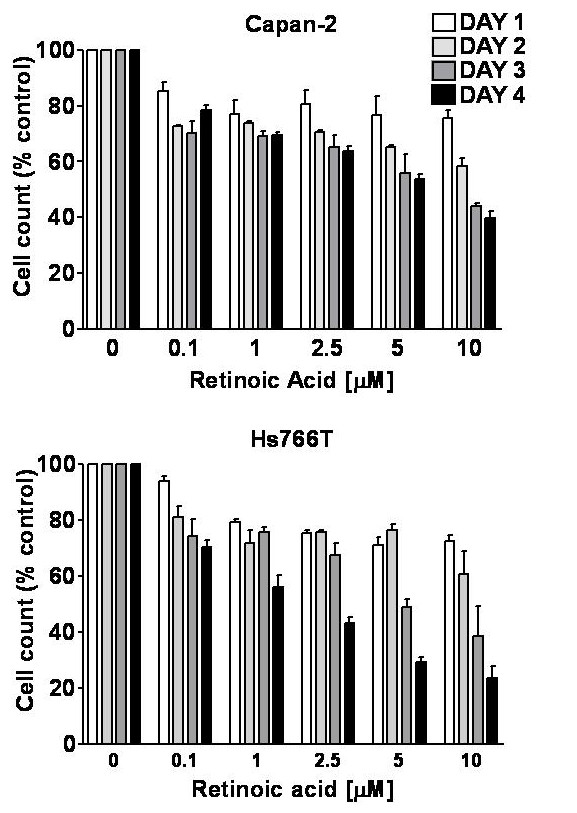
Effect of retinoic acid on the proliferation of human pancreatic cancer cells. All-trans-retinoic acid significantly inhibited the proliferation of CAPAN-2 (top panel) and Hs766T cells (bottom panel), in a concentration and time-dependent manner. Two-way ANOVA: Capan-2: Concentration effect *F*(3,48) = 46.6; Time effect *F*(5,48) = 147.8; Interaction *F*(15,48) = 5.79, all P < 0.0001; Hs766T: Concentration effect *F*(3,24) = 61.9; Time effect *F*(5,24) = 86.9; Interaction *F*(15,24) = 5.92, all P < 0.0001. Cell counts are expressed as percentage of control, bars represent means ± SEM.

**Figure 2:**
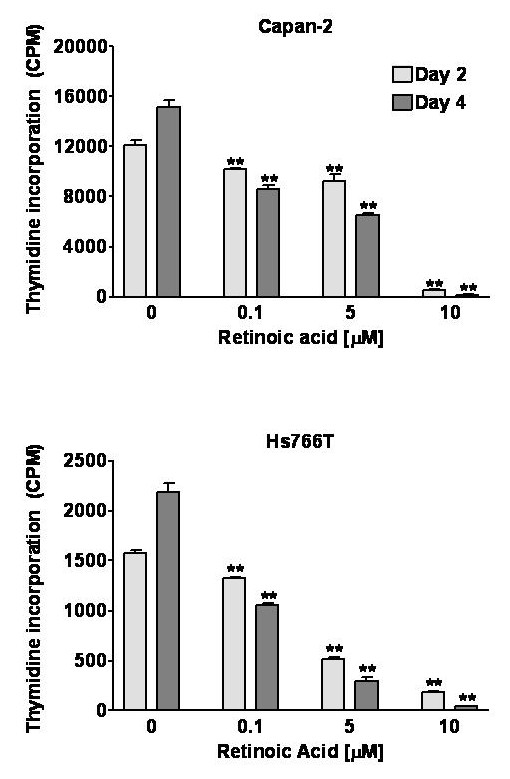
Effect of all-trans retinoic acid on incorporation of ^3^H-thymidine in CAPAN-2 (top panel) and Hs766T (bottom panel) cells over 48 and 96 hours. Concentration-dependent inhibition of DNA synthesis was observed in both cells lines. Two-way ANOVA: Capan-2: Concentration effect *F*(3,16) = 576, P < 0.0001; Time effect *F*(1,16) = 2.62, P = 0.125; Interaction *F*(3,16) = 28.9, P < 0.0001; Hs766T: Concentration effect *F*(3,24) = 846, P < 0.0001; Time effect *F*(1,24) = 0.05, P = 0.817; Interaction *F*(3,24) = 57.0, P < 0.0001. Data are expressed as counts per minute (CPM), bars represent means ± SEM.

Retinoic acid inhibited the growth of Capan-2 and Hs766T cells in a concentration and time-dependent manner. Cell counts measured as percent of control are shown in Figure 1. After 6 days of treatment, Hs766T and Capan-2 cell numbers were reduced to approximately 68% and 75% of control with 0.1 μM retinoic acid and to 20% and 32% of control in cells treated with the highest concentration (10 μM), respectively. Retinoic acid caused a similar reduction in ^3^H-thymidine incorporation in Capan-2 and Hs766T cells (Figure 2). On days 2 and 4, ^3^H-thymidine incorporation was virtually undetectable with 10 μM retinoic acid in both cell lines. A decrease in ^3^H-thymidine incorporation could be detected as early as 12 hours [9].

### Effect of conditioned media on the growth of in CCL-64 Cells

**Figure 3:**
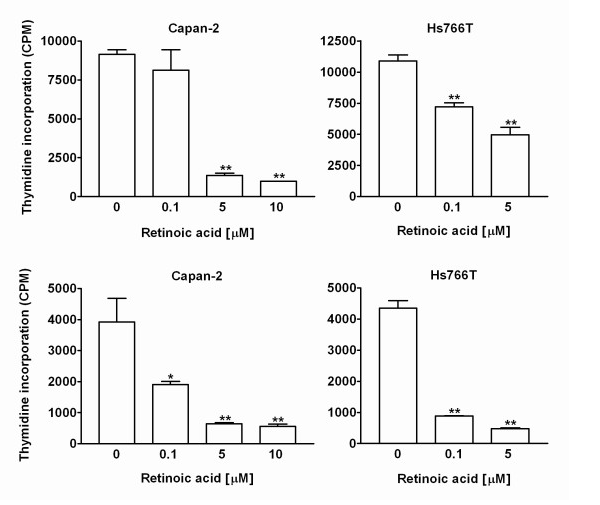
The effect of pancreatic cancer cell-conditioned media (CM) on ^3^H-thymidine incorporation by mink lung epithelial (CCL-64) cells. CCL-64 cells were exposed to non-activated CM (with only active TGFβ, top panels) and acid-activated CM (total TGFβ, bottom panels) from CAPAN-2 and Hs766T cells treated with retinoic acid for 48 hours. The growth of the CCL-64 cells was significantly attenuated by both types of CM. One way ANOVA: Capan-2, non-activated CM: *F*(3,11) = 40.6, P < 0.0001; Capan-2, activated CM: *F*(3,11) = 16.5, P = 0.0009; Hs766T, non-activated CM: *F*(2,11) = 39.2, P < 0.0001; Hs766T, activated CM: *F*(2,11) = 231, P < 0.0001. Data are expressed as counts per minute (CPM), bars represent means ± SEM. Significance of individual comparisons after Dunnett's multiple comparison test is indicated by ** = P < 0.01.

The conditioned media from Capan-2 and Hs766T cells treated with retinoic acid inhibited the growth of the CCL-64 cells in a concentration dependent manner, as shown in Figure 3. Significant growth inhibition of the CCL-64 cells was observed in both untreated {(active TGF-β) top panel} and acid activated {(total TGF-β) bottom panel} samples. In untreated samples a 1:2 dilution of media was the most effective, with a significant inhibition of growth at 5 and 10 μM retinoic acid in both cell lines. Marked growth inhibition of CCL-64 cells was seen at a 1:10 dilution of acid-activated conditioned media (data not shown).

### Effect of TGF-β immunoneutralization on CCL-64 cell proliferation in response to conditioned media from retinoic acid-treated pancreatic cancer cells

**Figure 4:**
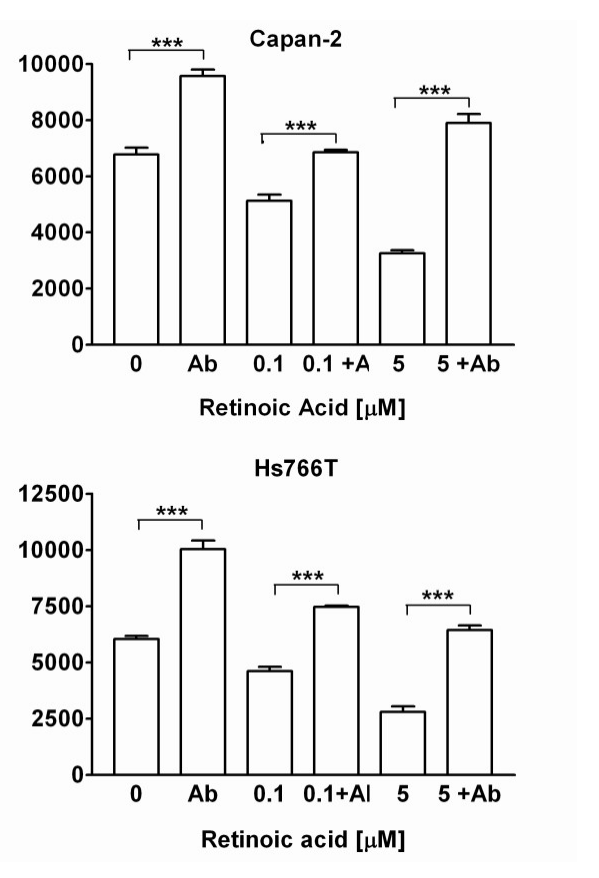
Effect of TGF-β pan-neutralizing antibody on inhibition of ^3^H-thymidine incorporation by CCL-64 cells. TGF-β antibody prevented growth inhibition of CCL-64 cells induced by CM from CAPAN-2 (top panel) and Hs766T (bottom panel) cells treated with 0.1 and 5 μM retinoic acid. One way ANOVA: Capan-2: *F*(5,17) = 101.9; HS766T: *F*(5,17) = 113.3, both P < 0.0001. Data are expressed as counts per minute (CPM), bars represent means ± SEM. Significance of individual comparisons after Bonferroni's multiple comparison test is indicated by *** = P < 0.001.

To confirm the specificity of the TGF-β bioassay in CCL-64 cells, immunoneutralization studies were carried out using different concentrations (25. 50 and 100 μg/ml) of a pan-specific TGF-β antibody. The results showed that 25 μg/ml of pan-specific antibody was sufficient to significantly reduce the growth inhibitory effects of the conditioned media on the CCL-64 cells. Furthermore, there was no significant difference between CCL-64 cells treated with 25, 50 and 100 μg/ml of the antibody in the conditioned media (data not shown). Therefore, subsequent studies with CCL-64 cells were conducted with 25 μg/ml antibody. The attribution of the stimulation of ^3^H-thymidine incorporation in CCL-64 cells by untreated and acid-treated conditioned media to the presence of TGF-β is supported by the observation that the stimulation was prevented by antibodies to TGF-β (Figure 4).

### Quantification of TGF-β2 mRNA

**Figure 5:**
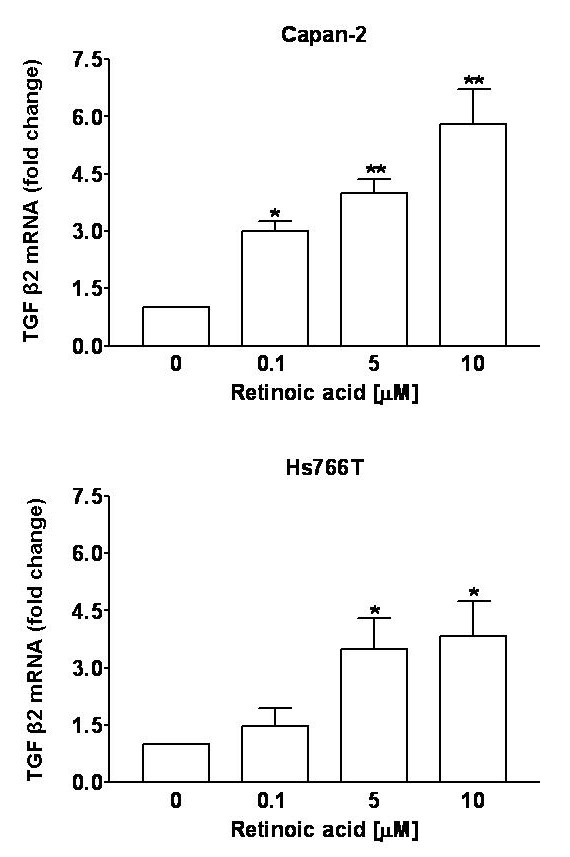
Effect of retinoic acid on TGF-β2 mRNA quantified by real-time RT-PCR in human pancreatic cancer cells. CAPAN-2 (top panel) and Hs766T cells (bottom panel) were treated with 0.1–10 μM retinoic acid for 18 hours. Retinoic acid caused a significant concentration-dependent increase in the TGF-β2 mRNA expression in these cells. Values were normalized to levels of mRNA for GAPDH, which was also used as a loading control. One way ANOVA: Capan-2: *F*(3,11) = 15.0, P = 0.0012; HS766T: *F*(3,11) = 4.72, P = 0.034. Data are expressed as fold-change from untreated cells, bars represent means ± SEM. Significance of individual comparisons after Dunnett's multiple comparison test is indicated by * = P < 0.05, ** = P < 0.01.

TGF-β2 mRNA expression was significantly increased by 4 and 6-fold after 18 hours of treatment with retinoic acid in Hs766T and Capan-2 cells, respectively (Figure 5). However at later time points such as 24 and 48 hours the expression levels were not significantly increased over control.

### Quantification of active and total TGF-β2 by enzyme-linked immunosorbent assay (ELISA)

**Figure 6:**
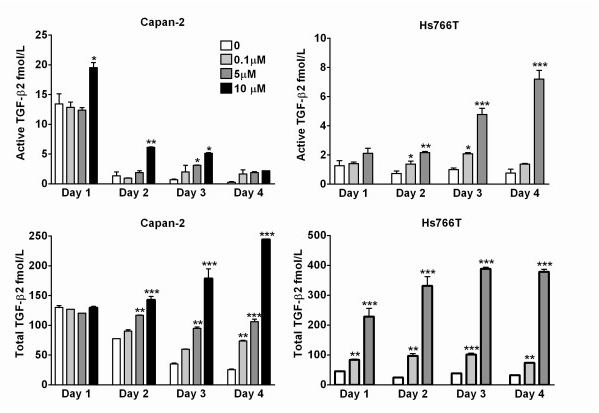
Effect of retinoic acid on secretion of TGF-β2 (active and total) by human pancreatic cancer cells measured by ELISA. Treatment with retinoic acid, stimulated secretion of active (top panels) and total (bottom panels) TGF-β2, from Capan-2 (left panels) and Hs766T cells (right panels), in a time and concentration-dependent manner Two-way ANOVA: Capan-2, active TGF-β2: Concentration effect *F*(3,16) = 36.4, P < 0.0001; Time effect *F*(3,16) = 356, P < 0.0001; Interaction *F*(9,16) = 4.47, P < 0.005; Capan-2, total TGF-β2: Concentration effect *F*(3,16) = 396; Time effect *F*(3,16) = 37.9; Interaction *F*(9,16) = 78.3, all P < 0.0001; Hs766T, active TGF-β2: Concentration effect *F*(2,17) = 155; Time effect *F*(3,17) = 27.4; Interaction *F*(6,17) = 27.8, all P < 0.0001. Hs766T, total TGF-β2: Concentration effect *F*(2,18) = 378, P < 0.0001; Time effect *F*(3,18) = 6.99, P = 0.0026; Interaction *F*(6,18) = 7.8, P = 0.0003. TGF-β2 concentrations are expressed as fmol/ml. Bars represent means ± SEM.

In a pilot study, active TGF-β2 concentrations were below the detection limit in the conditioned media from retinoic acid-treated pancreatic cancer cells. To bring the concentrations into the detectable range, the media was concentrated 10-fold for subsequent experiments. Retinoic acid treatment resulted in a significant concentration-dependent increase of both active and total TGF-β2 in the conditioned media from Capan-2 and Hs766T cells (Figure 6). These results clearly indicate that treatment of pancreatic cancer cells with retinoic acid significantly increases both the active and the precursor forms of TGF-β2 in a time and concentration dependent manner. Moreover, these results are also consistent with the bioassay data.

### Growth inhibition studies with TGF-β2

Based on the results from the ELISA, we determined a range for treatment of the pancreatic cancer cells with TGF-β2. This range (0.3–300 pM) spans the range seen for both active and total TGF-β2.

**Figure 7:**
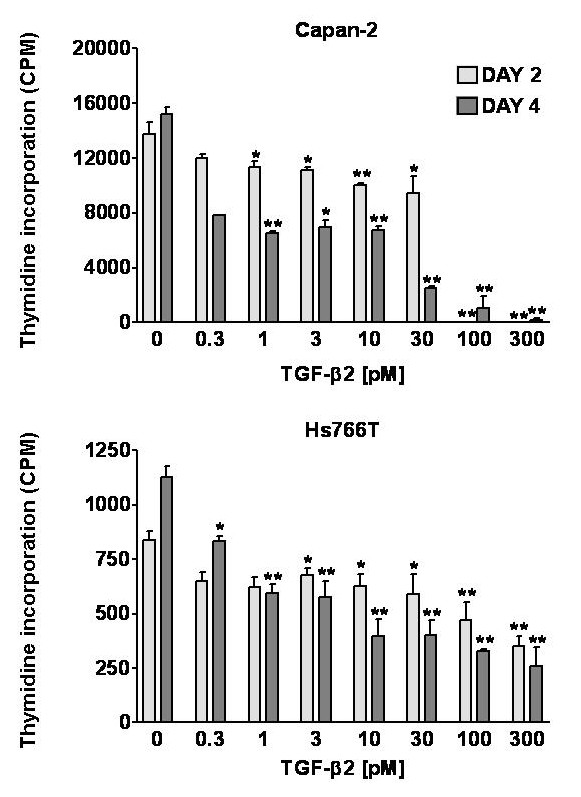
Effect of TGF-β2 on ^3^H-thymidine incorporation in human pancreatic cancer cells. Treatment of CAPAN-2 (top panel) and HS766T cells (bottom panel) with TGF-β2 significantly inhibited incorporation of ^3^H-thymidine in a concentration-dependent manner. A significant time-dependent effect was seen in Capan-2, but not Hs766T cells. Two-way ANOVA: Capan-2, Concentration effect *F*(7,32) = 181; Time effect *F*(1,32) = 104; Interaction *F*(7,32) = 18.2, all P < 0.0001; Hs766T: Concentration effect *F*(7,32) = 26.1, P < 0.0001; Time effect *F*(1,32) = 1.72, P = 0.199; Interaction *F*(7,32) = 4.96, P = 0.0007. Data are expressed in counts per minute (CPM). Bars represent means ± SEM.

Results of the growth inhibition studies with TGF-β2 are shown in Figure 7. Growth inhibition was studied by thymidine incorporation and cell counting (data not shown) and the results obtained with these two methods were comparable. After 4 days of treatment, 1 pM TGF-β2 significantly inhibited the growth of both cell lines. High concentrations (100–300 pM) caused cell death in Capan-2, while Hs766T cells were only growth inhibited at these concentrations. The TGF-β2 concentration of 1 pM falls within the active range of TGF-β2 measured by ELISA. These results indicate that the amount of active TGF-β2 secreted by the pancreatic cancer cells in response to retinoic acid is capable of mediating the growth inhibitory effects of the retinoic acid.

### Effect of TGF-β immunoneutralization on retinoic acid inhibited proliferation of pancreatic cancer cells

**Figure 8:**
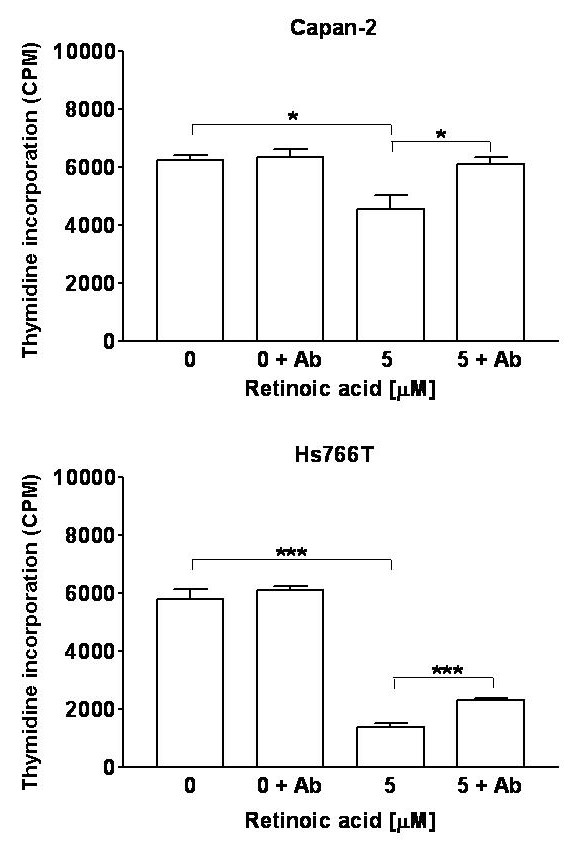
Effect of pan-neutralizing TGF-β antibody on retinoic acid-induced inhibition of ^3^H-thymidine incorporation in human pancreatic cancer cells. The TGF-β antibody prevented the growth inhibitory effects of retinoic acid in CAPAN-2 cells (top panel) and partially inhibited the effect in Hs766T cells (bottom panel). One way ANOVA: Capan-2: *F*(3,11) = 7.02, P = 0.0125; HS766T: *F*(3,11) = 136, P < 0.0001. Significance of individual comparisons after Bonferroni's multiple comparison test is indicated by * = P < 0.05, *** = P < 0.001. Data are expressed in counts per minute (CPM). Bars represent means ± SEM.

Treatment with the pan-neutralizing TGF-β antibody prevented the inhibitory effects of retinoic acid on of Capan-2 cells and partially prevented the effect in Hs766T cells (Figure 8).

## Discussion

In the present study, we have established a role of TGF-β2 in mediating the growth inhibitory effects of retinoic acid in human pancreatic adenocarcinoma cells. Retinoic acid increased the expression of TGF-β2 mRNA as well as the concentration of active and total TGF-β2. In addition to measurement by immunoassay, the biological activity TGF-β2 in the conditioned media of retinoid treated cells was evaluated by growth inhibition of CCL-64 mink lung epithelial cells. While this is a biologically relevant finding, it is neither quantitative nor specific for either TGF-β isoform. It is possible that a factor other than TGF-β may inhibit the growth of CCL-64 cells, however the bioassay is thought to be very specific for this peptide [34]. Furthermore, the specificity of TGF-β was confirmed using pan-TGF-β neutralizing antibodies, which prevented the growth inhibition of CCL-64 cells induced by the conditioned media. TGF-β2 levels were quantitatively assessed by ELISA. The disadvantage of ELISA is that the concentration of the peptide is determined by the epitope the antibody recognizes and may, therefore measure, non-biologically active form. However, in combination these assays indicate a significant increase in both the secreted latent and biologically active form of TGF-β from retinoid treated cells.

Retinoic acid stimulated secretion of latent and active TGF-β2 from Hs766T cells in a time and concentration-dependent manner. In contrast, while the TGF-β2 response in Capan-2 was concentration dependent, measured concentrations of active TGF-β2 decreased with time. Secreted TGF-β concentration measured on the second and fourth day should be higher than the first and third day, respectively. Since the media was changed at 48 and 96 hours, more TGF-β should accumulate by the second and fourth day. Therefore the lower levels seen at these time points must reflect a degree of degradation of TGF-β in the media. Thus the actual concentrations of TGF-β2 may be higher than those measured.

We have reported that cell lines that were previously shown to be resistant to the growth inhibitory effects of retinoic acid, were responsive when growth conditions were optimized. All isoforms of retinoic acid (9-cis, 13-cis and all trans) were equipotent in inducing growth inhibition in these cell lines [9]. Rosewicz et al showed that growth inhibition observed in Capan-2 cells in response to retinoic acid treatment correlated with decrease in PKC alpha expression levels [35]. Retinoic acid also induced differentiation at the morphological and biochemical levels in pancreatic cancer cells. Changes in morphology correlated with changes in metabolism such as increased carbonic anhydrase activity and mucin production [36,37]. In this study we quantified active TGF-β2 levels induced by retinoic acid in pancreatic cancer cells. We did not examine the effect of retinoic acid on TGF-β1 expression and activation because retinoic acid has no effect on the expression on TGF-β1 in pancreatic cancer cells [26]. The growth inhibitory effects of retinoic acid could also be partially mediated by p21^waf1 ^and TGF-β pathway. Since p21 mRNA and protein levels were found to be elevated by retinoic acid.

Retinoic acid induced TGF-β2 production in human adenocarcinoma cells and normal rat kidney fibroblasts [38]. We observed a transient increase in TGF-β2 mRNA in response to retinoic acid treatment followed by an increase in the active form of TGF-β2 as estimated by ELISA. This response can be transient since TGF-β is secreted in an inactive latent complex and is kept associated with the extracellular matrix. Retinoic acid can activate latent TGF-β2. Retinoic acid enhances the production of the tissue-type plasminogen activator which increases cell associated plasmin activity and activates latent TGF-β [27,28]. It is likely that a similar mechanism exists in pancreatic cancer cells. Retinoic acid can regulate the transcription of the TGF-β2 and TGF-β receptors, by the characteristic binding of its receptors to Sp1 and GC box motifs in the respective promoters. Retinoic acid treatment or RAR/RXR-overexpression strengthens the affinity of Sp1 to GC box motifs in the promoter by potentially inducing a conformational change in Sp1 thereby enhancing the transactivation of these genes [39].

Based on the results from the ELISA, we determined a range for treatment of the pancreatic cancer cells with TGF-β2. This range (0.3–300 pM) covers the expression for both active and total TGF-β2. Upon treating cancer cells with TGF-β2 we found that even the lower concentrations were sufficient to induce growth inhibition and cell death. These results indicate that the amounts of active TGF-β2 generated by the pancreatic cancer cells treated with retinoic acid are capable of mediating its growth inhibitory effects.

Immunoneutralization studies using pan-specific TGF-β antibody only partially blocked the growth inhibitory effects of retinoic acid in HS766T cells. There are several possible reasons for this; firstly, there could be insufficient antibody to neutralize the larger amount of TGF-β secreted from this cell line in response to 5 μM retinoic acid. Secondly, the antibody could bind to the large amount of latent TGF-β peptide, therefore making it unavailable for neutralizing the active TGF-β. Finally, TGF-β may be one of several factors involved in mediating the effects of retinoic acid.

The induction of latent and active TGF-β2 is independent of the DPC-4 status of the cells, since, activation was seen in cells with wild-type SMAD-4 (Capan-2) as well as in Hs766T cells, which have a homozygous deletion of this gene. Furthermore, both cell lines were sensitive to the growth inhibitory effects of TGF-β2, in a concentration and time-dependent manner. This indicates that SMAD independent pathways must be involved in mediating the growth inhibitory effects of TGF-β, at least in Hs766T cells. Growth inhibitory effects of TGF-β have been previously reported in SMAD-4 null pancreatic and colon cancer cells [16,19]. These SMAD-4 independent mechanisms of TGF-β signaling, in pancreatic cancer are not clearly understood. The activated TGF-β receptor complex activates several non SMAD-4 signaling pathways, including MAPK (p44/42 or ERK), PP2A/P70S6K, Rho A, TAK/MEKK1 [19] and phosphoinositide 3-kinase (PI3K) [11]. Further analysis of the molecular interactions mediating the responses to retinoic acid in SMAD-4 null cells will unravel the pathways involved. Since both cell lines have inactivating mutations in the p53^WAF-1 ^gene, the inhibitory effects of TGF-β in these cell lines are also clearly independent of p53^WAF-1 ^signaling.

## Conclusion

Retinoic acid induces growth inhibition in pancreatic cancer cells regardless of their degree of differentiation, k-ras, p53 and DPC-4 status [16,40]. Our current data is consistent with these previously published results. We have demonstrated in a step by step manner, that TGF-β is involved in mediating the growth inhibitory effects of retinoic acid. Smad-4 independent pathways are implicated in mediating the growth inhibitory effects of TGF-β in Hs766T cells. However, other factors are also likely to be involved. This is the first direct evidence of the involvement of TGF-β in mediating retinoic acid induced growth inhibition in pancreatic cancer. In conclusion, we have clearly demonstrated that the growth inhibitory effects of retinoic acid are partially, but significantly mediated through the increased expression of TGF-β2.

## Materials and methods

### Cell lines and treatment with retinoic acid

Well-differentiated (Capan-2) and poorly-differentiated (Hs766T) human pancreatic cancer cell lines were used for these studies. Both cell lines were grown as sub-confluent monolayer cultures in Dulbecco's modified Eagle's medium/Ham's F-12 medium (1:1) supplemented with 10% fetal calf serum, 100 units/ml penicillin and 100 μg/ml streptomycin. Cells were incubated at 37°C in a humidified atmosphere with 5% CO_2_. All experiments were carried out in the log phase of growth after the cells had been plated for 24 hours. A stock 100 mM solution of all-trans retinoic acid (Sigma Chemicals, St. Louis, MO) in DMSO was stored in 50 μl aliquots in dark brown tubes at -80°C. After dilution in medium, this stock gave final concentrations between 1 nM and 10 μM, with DMSO concentrations < 0.01%. All experiments with retinoic acid were carried out in subdued yellow light.

Cells were treated with retinoic acid in serum free conditions, using media containing a 1:1 mixture of DMEM and Ham's F-12 media, containing 4 mM L-glutamine, 1 mM pyruvate, 10 mM HEPES and 2.5 g/L glucose. Cell proliferation studies were conducted in 24-well plates. Media, with or without fresh retinoic acid were renewed every 48 hours.

### Cell counting and ^3^H-thymidine incorporation

Treated cells were washed with phosphate buffered saline (PBS) and detached using 0.25% trypsin solution containing 1 mM EDTA. Cells in suspension were counted using an automated particle counter (Model Z1, Coulter Electronics).

After treatment with retinoic acid or medium the cells were washed with PBS and incubated with ^3^H-thymidine (1 μCi/well, 25 Ci/mmol, Amersham Life Science, Arlington Heights, IL) in serum free medium for 2 hours. The medium was removed and the cells were washed three times with PBS. Cellular proteins were then precipitated twice with ice cold 10% TCA for 15 minutes, to remove acid soluble materials including free thymidine. The precipitates were solubilized in 1.0 M NaOH and counted in a liquid scintillation counter.

### Measuring bioactivity of TGF-β in conditioned medium using CCL-64 cells

To measure the bioactivity of secreted TGF-β, mink lung epithelial (CCL-64) cells were treated with the conditioned medium from retinoic acid-treated and untreated control cells. To prepare conditioned medium, cancer cells were treated with 0.1, 5 and 10 μM retinoic acid for 72 hours, washed twice with PBS, and incubated in serum free fresh medium containing 0.1% BSA for 24 hours. Conditioned medium was then collected in siliconized tubes and aliquots were stored at -20°C. Repeated freezing and thawing was avoided. For bioassay of active TGF-β, CCL-64 cells were seeded in 10% FBS containing MEM medium in 24 well plates (20,000 cells/well) for 24 hours and then cultured in serum-free medium for 24 hours. Cells were plated in triplicate for each concentration of retinoic acid. CCL-64 cells were incubated for 24 hours in CM. The proliferation of CCL-64 cells was measured by inhibition of ^3^H-thymidine incorporation as described above.

To demonstrate the presence of total TGF-β (latent and active), diluted 1:2 conditioned media (1 ml) was added directly or following acidification to pH 2.0 with 1N HCl, incubation at 20°C for 20 minutes and readjustment of the pH to 7.4 with 1N NaOH, to the plated CCL-64 cells.

### RNA preparation from pancreatic cancer cells

Total RNA preparations from retinoic acid treated pancreatic cancer cells was carried out using the total RNA miniprep kit (DMN10, Sigma, St. Louis, MO). Pancreatic cancer cells grown in T-25 cm^2 ^tissue culture flasks were washed twice with PBS and lysed with the lysis buffer containing guanidine thiocyanate and 2-mercaptoethanol to release the RNA and inactivate the RNases. Lysates were spun through a filtration column to remove cellular debris and shear DNA. The filtrate was then applied to a silica column and washed and the RNA eluted according to the manufacturer's instructions. The RNA was then treated with 1 μl DNase (Ambion, Austin, TX). The quality and the concentration of the RNA were determined spectrophotometrically.

### Preparation of cDNA by reverse-transcription

The volume corresponding to 1 μg total RNA from retinoic acid treated and untreated pancreatic cancer cells was mixed with 1 μl of oligo dT primers (Invitrogen, Carlsbad, CA) and incubated at 68–70°C for 10 mins and then put on ice to separate the RNA molecules. A master-mix was then prepared using 5× first-strand buffer, 2'-deoxynucleoside 5'-triphosphate (dNTP) (10 mM), ribonuclease (RNase) inhibitor (25 U) and Moloney Murine leukemia virus reverse transcriptase all purchased from Invitrogen (Carlsbad, CA). Total reaction volume (25 μl) was then subjected to RT-PCR (42°C-60 minutes, 95°C-5 minutes, 4°C-5 minutes). The resulting complementary DNA (cDNA) was stored at -20°C.

### Real-time PCR

The cDNA (40 ng) obtained from reverse transcription was subjected to quantitative real-time polymerase chain reaction (RT-PCR) using the SYBER Green detection system (Applied Biosystems, Foster City, CA). GAPDH from control and treated samples was used as a loading control and also to normalize the transcripts, since GAPDH transcripts are unaltered in response to retinoic acid. The RT-PCR forward and reverse primers for TGF-β2 and GAPDH were as follows:

TGF-β2 forward:GGGTGGAAATGGATACACGAA

TGF-β2 reverse:AGGACCCTGCTGTGCTGAGT

GAPDH forward:TGGGCTACACTGAGCACCAG

GAPDH reverse: GGGTGTCGCTGTTGAAGTCA

### Quantification of secreted TGF-β2 by ELISA

Quantification of both the active and latent forms of TGF-β2 was carried out using the TGF-β2 E_max_™Enzyme-Linked Immunosorbent Assay system (Promega, Madison, WI). Capan-2, and Hs766T cells were treated with retinoic acid for 24, 48, 72 and 96 hours, conditioned media were collected as described above. For assaying active TGF-β2, samples were concentrated 10-fold by the freeze drying and reconstituting in one-tenth of the original volume in "sample buffer" (provided in the assay kit). To study the expression of total TGF-β2, samples were acid activated as described above. For consistency, the TGF-β2 ELISA and bioassay with CCL-64 cells were carried out at the same time with the same samples.

### Cell growth studies with TGF-β2

TGF-β2 (R&D Systems, Minneapolis, MN) was dissolved in 100 mM formic acid with 0.1% BSA and 50 mg/ml lactose. The solution was divided into aliquots and freeze dried. Upon reconstitution in 1 ml of media the TGF-β2 concentration was 200 nM. The cells were then treated with different concentrations of TGF-β2, which includes the concentration range in conditioned media from the three cell lines, 0.3–300 pM. Thymidine incorporation was measured as described above.

### Immunoneutralization studies

To confirm the presence of TGF-β in the conditioned media, mediating the growth inhibitory effects on the CCL-64 cells, immunoneutralization studies were carried out using different concentrations (25, 50 and 100 μg/ml) of a pan-specific TGF-β antibody. CCL-64 cells were treated with cancer cell conditioned media diluted 1:2, with or without the pan-neutralizing TGF-β antibody as previously described (R&D Systems, Minneapolis, MN) [34]. Cell growth was measured by ^3^H-thymidine incorporation as described above.

### Statistical analysis

Data was analyzed by one way analysis of variance (ANOVA) with Dunnett's post test for multiple comparisons against control and Bonferroni's test for pre-selected comparisons between groups. Two-way ANOVA was also performed for experiments studying the effects of different treatments over time.

## Competing interests

The author(s) declare that they have no competing interests.

## Authors' contributions

BS was involved with experimental design, all the experimental procedures, data interpretation and drafting of the manuscript, RFM, RHB and TEA were involved in the conception and design of the studies, manuscript preparation graphics and statistical analysis, XD and ABR were involved with study design, and contributed to the real-time RT-PCR, cell growth, immunoneutralization and ELISA studies. All authors approve the final version of the manuscript for publication.

## References

[B1] Jemal A, Murray T, Samuels A, Ghafoor A, Ward E, Thun MJ (2003). Cancer statistics, 2003. CA Cancer J Clin.

[B2] Cowgill SM, Muscarella P (2003). The genetics of pancreatic cancer. Am J Surg.

[B3] Marill J, Idres N, Capron CC, Nguyen E, Chabot GG (2003). Retinoic acid metabolism and mechanism of action: a review. Curr Drug Metab.

[B4] Altucci L, Gronemeyer H (2001). The promise of retinoids to fight against cancer. Nat Rev Cancer.

[B5] Warrell RP, de The H, Wang ZY, Degos L (1993). Acute promyelocytic leukemia. N Engl J Med.

[B6] Berg WJ, Divgi CR, Nanus DM, Motzer RJ (2000). Novel investigative approaches for advanced renal cell carcinoma. Semin Oncol.

[B7] Reynolds CP, Lemons RS (2001). Retinoid therapy of childhood cancer. Hematol Oncol Clin North Am.

[B8] Mangelsdorf DJ, Evans RM (1995). The RXR heterodimers and orphan receptors. Cell.

[B9] El-Metwally TH, Adrian TE (1999). Optimization of treatment conditions for studying the anticancer effects of retinoids using pancreatic adenocarcinoma as a model. Biochem Biophys Res Commun.

[B10] Pettersson F, Dalgleish AG, Bissonnette RP, Colston KW (2002). Retinoids cause apoptosis in pancreatic cancer cells via activation of RAR-gamma and altered expression of Bcl-2/Bax. Br J Cancer.

[B11] Massague J, Blain SW, Lo RS (2000). TGFbeta signaling in growth control, cancer, and heritable disorders. Cell.

[B12] Yue J, Mulder KM (2001). Transforming growth factor-beta signal transduction in epithelial cells. Pharmacol Ther.

[B13] Tsukazaki T, Chiang TA, Davison AF, Attisano L, Wrana JL (1998). SARA, a FYVE domain protein that recruits Smad2 to the TGFbeta receptor. Cell.

[B14] Derynck R, Zhang Y, Feng XH (1998). Smads: transcriptional activators of TGF-beta responses. Cell.

[B15] Hahn SA, Schutte M, Hoque AT, Moskaluk CA, da Costa LT, Rozenblum E, Weinstein CL, Fischer A, Yeo CJ, Hruban RH, Kern SE (1996). DPC4, a candidate tumor suppressor gene at human chromosome 18q21.1. Science.

[B16] Dai JL, Schutte M, Bansal RK, Wilentz RE, Sugar AY, Kern SE (1999). Transforming growth factor-beta responsiveness in DPC4/SMAD4-null cancer cells. Mol Carcinog.

[B17] Fink SP, Swinler SE, Lutterbaugh JD, Massague J, Thiagalingam S, Kinzler KW, Vogelstein B, Willson JK, Markowitz S (2001). Transforming growth factor-beta-induced growth inhibition in a Smad4 mutant colon adenoma cell line. Cancer Res.

[B18] Sirard C, Kim S, Mirtsos C, Tadich P, Hoodless PA, Itie A, Maxson R, Wrana JL, Mak TW (2000). Targeted disruption in murine cells reveals variable requirement for Smad4 in transforming growth factor beta-related signaling. J Biol Chem.

[B19] Derynck R, Zhang YE (2003). Smad-dependent and Smad-independent pathways in TGF-beta family signalling. Nature.

[B20] Dickens TA, Colletta AA (1993). The pharmacological manipulation of members of the transforming growth factor beta family in the chemoprevention of breast cancer. Bioessays.

[B21] Morales TI, Roberts AB (1992). The interaction between retinoic acid and the transforming growth factors-beta in calf articular cartilage organ cultures. Arch Biochem Biophys.

[B22] Lardon F, Snoeck HW, Haenen L, Lenjou M, Nijs G, Weekx SF, Van Ranst PC, Berneman ZN, Van Bockstaele DR (1996). The combined effects of all-trans retinoic acid and TGF-beta on the initial proliferation of normal human bone marrow progenitor cells. Leukemia.

[B23] Kishi H, Kuroda E, Mishima HK, Yamashita U (2001). Role of TGF-beta in the retinoic acid-induced inhibition of proliferation and melanin synthesis in chick retinal pigment epithelial cells in vitro. Cell Biol Int.

[B24] Valette A, Botanch C (1990). Transforming growth factor beta (TGF-beta) potentiates the inhibitory effect of retinoic acid on human breast carcinoma (MCF-7) cell proliferation. Growth Factors.

[B25] Behbakht K, DeGeest K, Turyk ME, Wilbanks GD (1996). All-trans-retinoic acid inhibits the proliferation of cell lines derived from human cervical neoplasia. Gynecol Oncol.

[B26] Choudhury A, Singh RK, Moniaux N, El-Metwally TH, Aubert JP, Batra SK (2000). Retinoic acid-dependent transforming growth factor-beta 2-mediated induction of MUC4 mucin expression in human pancreatic tumor cells follows retinoic acid receptor-alpha signaling pathway. J Biol Chem.

[B27] Glick AB, Flanders KC, Danielpour D, Yuspa SH, Sporn MB (1989). Retinoic acid induces transforming growth factor-beta 2 in cultured keratinocytes and mouse epidermis. Cell Regul.

[B28] Kojima S, Rifkin DB (1993). Mechanism of retinoid-induced activation of latent transforming growth factor-beta in bovine endothelial cells. J Cell Physiol.

[B29] Imai S, Okuno M, Moriwaki H, Muto Y, Murakami K, Shudo K, Suzuki Y, Kojima S (1997). 9,13-di-cis-Retinoic acid induces the production of tPA and activation of latent TGF-beta via RAR alpha in a human liver stellate cell line, LI90. FEBS Lett.

[B30] Danielpour D (1996). Induction of transforming growth factor-beta autocrine activity by all-trans-retinoic acid and 1 alpha,25-dihydroxyvitamin D3 in NRP-152 rat prostatic epithelial cells. J Cell Physiol.

[B31] Batova A, Danielpour D, Pirisi L, Creek KE (1992). Retinoic acid induces secretion of latent transforming growth factor beta 1 and beta 2 in normal and human papillomavirus type 16-immortalized human keratinocytes. Cell Growth Differ.

[B32] Borger DR, Mi Y, Geslani G, Zyzak LL, Batova A, Engin TS, Pirisi L, Creek KE (2000). Retinoic acid resistance at late stages of human papillomavirus type 16-mediated transformation of human keratinocytes arises despite intact retinoid signaling and is due to a loss of sensitivity to transforming growth factor-beta. Virology.

[B33] Defacque H, Piquemal D, Basset A, Marti J, Commes T (1999). Transforming growth factor-beta1 is an autocrine mediator of U937 cell growth arrest and differentiation induced by vitamin D3 and retinoids. J Cell Physiol.

[B34] Garrigue-Antar L, Barbieux I, Lieubeau B, Boisteau O, Gregoire M (1995). Optimisation of CCL64-based bioassay for TGF-beta. J Immunol Methods.

[B35] Rosewicz S, Brembeck F, Kaiser A, Marschall ZV, Riecken EO (1996). Differential growth regulation by all-trans retinoic acid is determined by protein kinase C alpha in human pancreatic carcinoma cells. Endocrinology.

[B36] El-Metwally TH, Hussein MR, Pour PM, Kuszynski CA, Adrian TE (2005). High concentrations of retinoids induce differentiation and late apoptosis in pancreatic cancer cells in vitro. Cancer Biol Ther.

[B37] El-Metwally TH, Hussein MR, Abd-El-Ghaffar SK, Abo-El-Naga MM, Ulrich AB, Pour PM (2006). Retinoic acid can induce markers of endocrine transdifferentiation in pancreatic ductal adenocarcinoma: preliminary observations from an in vitro cell line model. J Clin Pathol.

[B38] Danielpour D, Kim KY, Winokur TS, Sporn MB (1991). Differential regulation of the expression of transforming growth factor-beta s 1 and 2 by retinoic acid, epidermal growth factor, and dexamethasone in NRK-49F and A549 cells. J Cell Physiol.

[B39] Shimada J, Suzuki Y, Kim SJ, Wang PC, Matsumura M, Kojima S (2001). Transactivation via RAR/RXR-Sp1 interaction: characterization of binding between Sp1 and GC box motif. Mol Endocrinol.

[B40] Sipos B, Moser S, Kalthoff H, Torok V, Lohr M, Kloppel G (2003). A comprehensive characterization of pancreatic ductal carcinoma cell lines: towards the establishment of an in vitro research platform. Virchows Arch.

